# Unraveling Energy Storage Performance and Mechanism of Metal–Organic Framework‐Derived Copper Vanadium Oxides with Tunable Composition for Aqueous Zinc‐Ion Batteries

**DOI:** 10.1002/smtd.202400819

**Published:** 2024-09-17

**Authors:** Ashok Kumar Kakarla, Hari Bandi, Wasim Akram Syed, D. Narsimulu, R. Shanthappa, Jae Su Yu

**Affiliations:** ^1^ Department of Electronics and Information Convergence Engineering, Institute for Wearable Convergence Electronics Kyung Hee University 1732 Deogyeong‐Daero, Giheung‐gu Yongin‐si Gyeonggi‐do 17104 Republic of Korea

**Keywords:** aqueous zinc‐ion batteries, copper vanadium oxides, metal–organic frameworks, synergistic energy storage mechanism

## Abstract

Achieving high‐performance aqueous zinc (Zn)‐ion batteries (AZIBs) requires stable and efficient cathode materials capable of reversible Zn‐ion intercalation. Although layered vanadium oxides possess high Zn‐ion storage capacity, their sluggish kinetics and poor conductivity present significant hurdles for further enhancing the performance of AZIBs. In response to this challenge, a dissolution‐regrowth and conversion approach is formulated using metal–organic frameworks (MOFs) as a sacrificial template, which enables the in situ creation of copper vanadium oxides (CuVO_x_) with porous 1D channels and distinctive nanoarchitectures. Owing to their distinctive structure, the optimized CuVO_x_ cathode experiences a reaction involving the synergistic insertion/extraction of Zn^2+^, resulting in rapid Zn^2+^ diffusion kinetics and enhanced electrochemical activity postactivation. Specifically, the activated electrode delivers a reversible capacity of 519 mAh g^−1^ at 0.5 A g^−1^ for AZIBs. It is noteworthy that the electrode exhibits a remarkable reversible rate capacity of 220 mAh g^−1^ at 5 A g^−1^ with excellent durable cycleability, retaining 88% of its capacity even after 3000 cycles. Various ex situ testing methods endorse the reversible insertion/extraction of Zn^2+^ in the CuVO_x_ cathode. This study provides a novel insight into high‐performance MOF‐derived unique structure designs for AZIB electrodes.

## Graphical abstract

To enhance aqueous zinc‐ion batteries (AZIBs), metal‐organic framework (MOF)‐derived copper vanadium oxides (CuVO_x_) with porous 1D channels are developed, improving Zn^2+^ diffusion and electrochemical activity. The optimized CuVO_x_ cathode delivers 519 mAh g^−1^ at 0.5 A g^−1^, with excellent cycling stability, retaining 88% capacity after 3000 cycles. This study offers insights into advanced MOF‐derived cathode designs for high‐performance AZIBs.

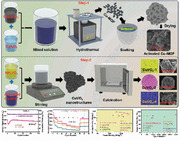

## 1 Introduction

The proliferation of portable electronic devices has spurred the demand for lightweight, flexible, and easily transportable energy storage solutions. Among these options, lithium (Li)‐ion batteries (LIBs) stand out because of their exceptional energy density, making them the preferred power source for a wide range of applications.^[^ ^1^, ^2^ ^]^ Nonetheless, there is a rising need for safer energy storage solutions due to the scarcity of Li resources, inevitable safety risks, and environmental issues. As a result, aqueous zinc (Zn)‐ion batteries (AZIBs) have garnered significant attention for large‐scale energy storage, thanks to their high theoretical capacity (820 mAh g^−1^), low redox potential (−0.76 V vs standard hydrogen electrode), cost‐effective Zn anode, excellent safety profile, and eco‐friendly replacement for LIBs.^[^ ^3^, ^4^, ^5^, ^6^, ^7^ ^]^ Even though AZIBs have several advantages, the cathode material is a crucial component that restricts their performance. In the realm of AZIB research, creating high‐performance cathode materials has become a critical challenge.^[^ ^8^, ^9^ ^]^ So far, the widely explored cathode materials for AZIBs are manganese (Mn)‐ and vanadium (V)‐based materials, organic molecules, and Prussian blue analogs (PBAs).^[^ ^10^ ^]^ The cathode materials of AZIBs are beset by either unsatisfactory Zn^2+^ storage capacity or poor cycling performance due to the sluggish diffusion kinetics of Zn^2+^, dissolution, or volume expansion with repeated insertion/extraction of Zn^2+^. Generally, Mn‐based cathodes exhibit significant theoretical capacity and high operating voltage but suffer from inferior cycle performance. On the other hand, PBAs have high voltage and reversibility, but the capacity (typically below 100 mAh g^−1^) is unsatisfactory.^[^ ^11^ ^]^ Particularly, the utilization of V‐based materials as active cathodes in AZIBs has attracted significant interest, primarily due to their large specific capacity caused by multivalent redox reactions (V^3+^↔V^4+^↔V^5+^). However, many V‐based cathodes face challenges associated with structural deterioration caused by V‐dissolution in aqueous electrolytes. To address this issue, strategies such as interlayer engineering, metal/nonmetal ion doping, and electrolyte optimization have been explored, especially concentrating on the stabilization of the V_2_O_5_ and V_3_O_8_ type crystal structures. These approaches aim to enhance Zn^2+^ storage performance by mitigating diffusion barriers and blocking the intercalation pathway for water‐related species.^[^ ^12^, ^13^ ^]^ Although wide research efforts concentrated on advancing high‐performance V‐based cathodes, the rational composition of unique nanostructured V‐oxides remains a challenge. Such composition is important for reaching enhanced electrochemical performance in Zn^2+^ storage applications.

Over the past 10 years, metal–organic frameworks (MOFs) have received substantial consideration in energy storage fields, such as LIBs,^[^ ^14^ ^]^ AZIBs,^[^ ^15^ ^]^ supercapacitors,^[^ ^16^, ^17^ ^]^ and other new energy storage devices,^[^ ^18^ ^]^ due to the advantages of superior surface area, structural diversity, and tunable frameworks. However, the majority of MOF materials have miserable structural solidity and conductivity, which is why they typically serve as base materials for high‐temperature pyrolysis to produce porous carbon, derived metal oxides, sulfides, nitrates, phosphides, and composites. In addition to this, the porous structure of MOF‐derived materials is usually occluded, causing the “dead mass,” which is unavailable for Zn^2+^ diffusion. Newly, Tong et al.^[^ ^19^ ^]^ developed MOF‐derived heterostructured C@VO_2_@V_2_O_5_ for high‐performance and stable cathode material for AZIBs. Subsequently, other investigators/researchers have revealed that Zn^2+^ may be retained in replacement of MOFs‐derived oxide cathodes, mostly using cobalt‐ or Mn‐based MOF materials. These materials’ distinct structures and inherent morphologies imply potential development in AZIBs.^[^ ^20^, ^21^, ^22^ ^]^ However, there are limited reports on the development of robust cathode materials for AZIBs utilizing porous copper (Cu)‐based MOFs as templates. Zeolitic imidazolate frameworks containing imidazolate linkers have gained significant attention due to their extensive surface area, high porosity, and ease of functionalization.^[^ ^23^ ^]^ Specifically, Cu‐MOF (Cu(II)) emerges as a promising alternative owing to its high adsorption capacity, cost‐effectiveness, and thermal stability in aqueous environments, aligning well with the operational conditions of AZIBs. For this reason, there is a strong desire to leverage MOFs as precursors and templates for designing functional materials with adjustable composition and distinctive morphologies.

By taking the benefits of MOFs, herein, we accomplish the target mentioned above by exploring the electroactive function of V species into the Cu‐MOF to generate the heterogeneous porous structures that could provide pathways, allowing fast Zn^2+^ extraction/insertion transport. By employing a MOF‐template approach, three types of copper vanadium oxides (CuVO_x_) with different morphologies were controllably obtained by precisely tuning the amount of V precursor, which efficiently offers a reversible Zn storage but produces a remarkable difference in performance. As a result, the optimized CuVO_x_ nanoparticles (NPs) cathode coupled with the Zn anode delivered a stable capacity with superior rate capability and excellent long‐term stability. Moreover, a typical consequence of fast Zn‐ion kinetics accompanied by high structural stability during cycling was demonstrated on the CuVO_x_ NPs electrode.

## 2 Results and Discussion

### 2.1 Morphological and Phase Properties of the As‐Synthesized Materials

**Figure 1:**
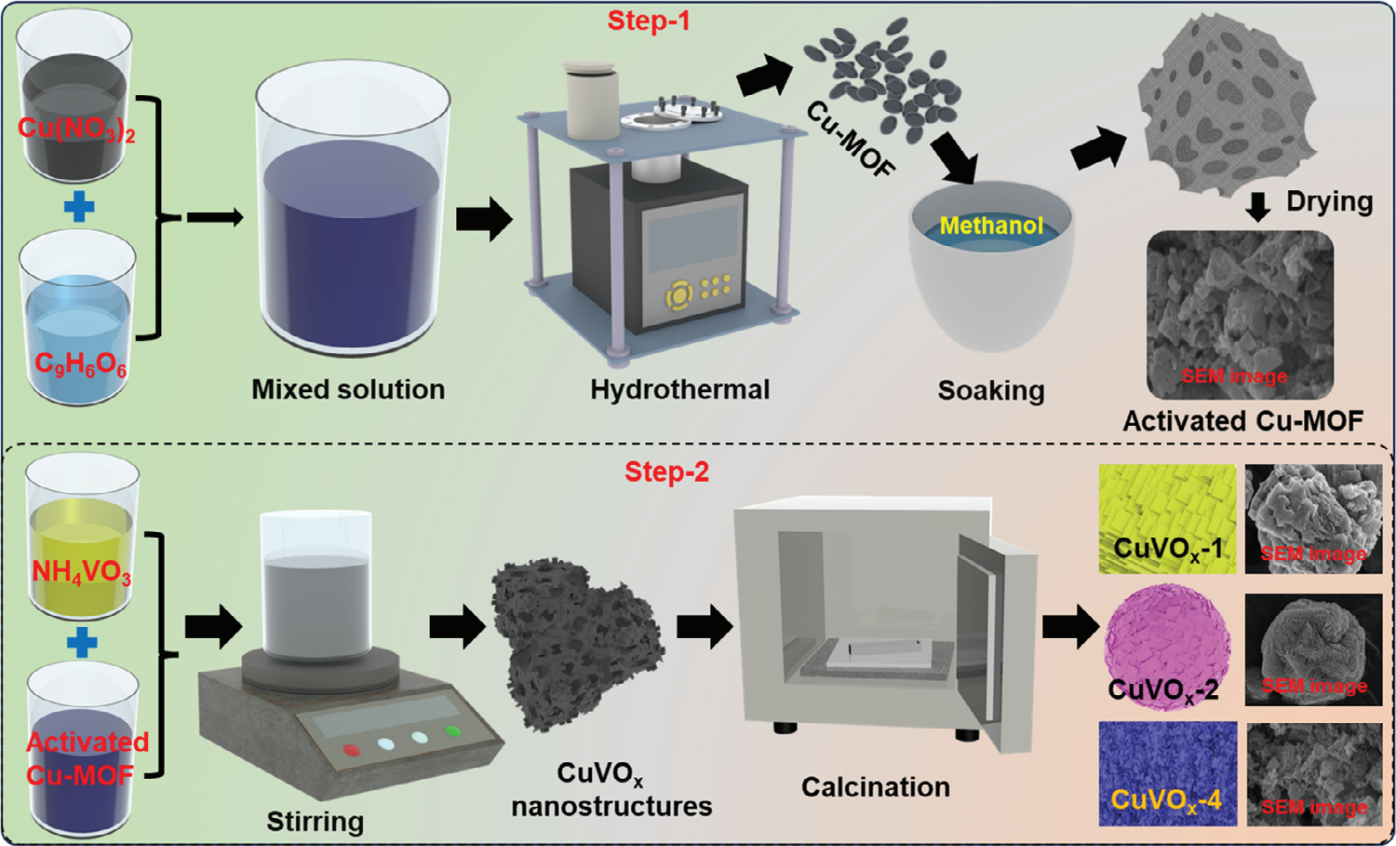
Schematic illustration for the conversion of Cu‐MOF to CuVO_x_ with different morphologies.

A template reaction was utilized to synthesize CuVO_x_ (CuVO_x_‐1, CuVO_x_‐2, and CuVO_x_‐4) species with diverse nanostructures by varying the concentration of the V source, as depicted in **Figure** **1**. Trimesic acid served as the organic linker because of its ability to interact with metal ions such as Cu. Initially, Cu‐MOF nanoplates were fabricated via a straightforward hydrothermal method. The adaptability of Cu‐MOF properties, including stability and functionality, allows customization to meet specific energy storage requirements. Furthermore, the activation of Cu‐MOF using methanol offers the advantage of increasing porosity and surface area, enhancing its adsorption capacity and catalytic activity across various applications. Additionally, this activation process is performed in the extraction of guest molecules surrounded by the MOF during synthesis, ensuring optimal sample performance. Subsequently, V species and Cu‐MOF were combined in an aqueous solution, where OH^−^ ions liberated from an ammonia solution at high temperatures slowly consumed Cu^2+^ ions in Cu‐MOF, resulting in the formation of notched‐like NPs with a unique template that offers a versatile approach for creating CuVO_x_ nanostructures with diverse morphologies.

**Figure 2:**
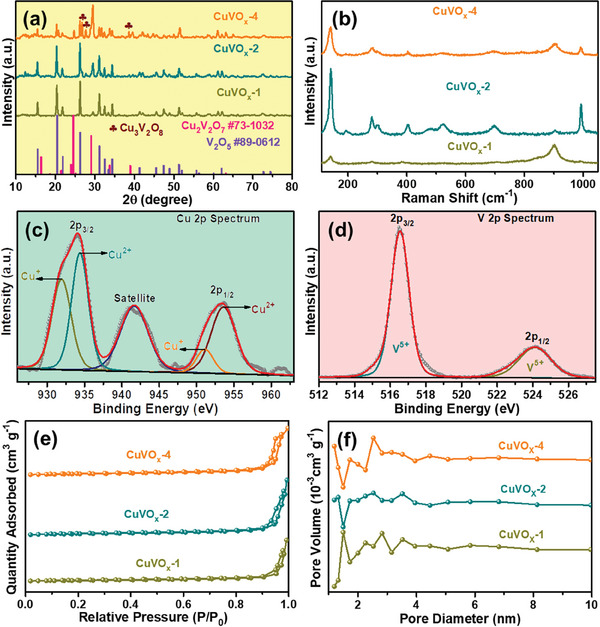
a) XRD patterns and b) Raman spectra of the as‐prepared CuVO_x_ materials. XPS spectra of c) Cu 2p and d) V 2p for the CuVO_x_‐2 material. e) N_2_ adsorption‐desorption isotherms and f) pore size distributions of the as‐prepared CuVO_x_ materials.

The aforementioned phenomena, particularly the presence of increased V species, in conjunction with the role of the Cu‐MOF template, are hypothesized to be the key underlying mechanisms driving the formation of these unique porous structures. The composition and crystallinity of CuVO_x_ were utterly investigated through X‐ray diffraction (XRD) measurements, as depicted in **Figure** **2**a. Upon analysis, it was observed that the CuVO_x_‐1 and CuVO_x_‐2 samples exhibited similar diffraction patterns with characteristic peaks at the 2θ values of 15.3°, 20.2°, 21.6°, 26.0°, 30.9°, 32.3°, 33.2°, 34.2°, 41.1°, 45.3°, 47.2°, 48.7°, 51.1°, 51.8°, 55.5°, 56.1°, 59.8°, 61.9°, 72.5°, and 74.2°. These peaks correspond to the crystalline planes of (200), (001), (101), (110), (301), (011), (111), (310), (002), (411), (600), (012), (020), (601), (021), (121), (701), (710), (403), and (313), providing insights into the crystal structure of the synthesized materials, which can be ascribed to the orthorhombic phase of V_2_O_5_ (#89‐0612, pmmn, 59). Additionally, other peaks observed at 16.5°, 18.7°, 21.5°, 24.1°, 24.7°, 25.2°, 29.2°, 33.8°, 39.1°, 51.4°, and 63.3° are well matched with the monoclinic phase of Cu_2_V_2_O_7_ (#73‐1032). The presence of these distinct diffraction peaks confirms the formation of CuVO_x_ with specific crystallographic orientations. Furthermore, the CuVO_x_‐4 sample exhibited a relatively lower degree of crystallinity compared to the CuVO_x_‐1 and CuVO_x_‐2 samples. The XRD pattern of the CuVO_x_‐4 sample revealed diffraction peaks at the 2θ values of 26.8°, 27.4°, and 38.7°, corresponding to the (021), (111), and (102) planes of Cu_3_V_2_O_8_, respectively. These results indicate the formation of Cu_3_V_2_O_8_ with distinct crystal structures, highlighting the influence of synthesis parameters on the final composition and crystallinity of the synthesized materials. In the Raman spectra (Figure 2b), the distinct vibrational bands of CuVO_x_ samples suggest a complex composition involving a mixed phase of V_2_O_5_ and Cu_2_V_2_O_7_. Notably, the prominent peaks at 139.9, 281.13, 403.04, 698.17, and 992.55 cm^−1^ correspond to the reported vibrational modes of V_2_O_5_, indicating their presence within the CuVO_x_.^[^ ^24^, ^25^ ^]^ In addition, characteristic features at 194, 301, and 943 cm^−1^ are indicative of Cu_2_V_2_O_7_.^[^ ^26^ ^]^ This spectral analysis underscores the nuanced structural variations among the synthesized CuVO_x_ materials, providing valuable insights into their composition and potential electrochemical behavior. Furthermore, the inductively coupled plasma (ICP) spectroscopy analysis was used to determine the elemental composition of the different CuVO_x_ samples, revealing significant variations in Cu:V ratios (Figure S1, Supporting Information). CuVO_x_‐1 has Cu content of 33.95% and V content of 66.05% (Cu:V ratio ≈1:1.94), CuVO_x_‐2 shows 18.28% Cu and 81.72% V (Cu:V ratio ≈1:4.47), and CuVO_x_‐4 has 6.87% Cu and 93.13% V (Cu:V ratio ≈1:13.56). The reduced Cu content and increased V dominance in CuVO_x_‐2 could impact the material's crystal structure, ion mobility, and electrochemical properties, particularly influencing capacity, stability, and conductivity. The shift in Cu:V ratios highlights CuVO_x_‐2 as a key sample for further investigation due to its potential to enhance Zn storage and overall battery performance.

The oxidation state and chemical composition of CuVO_x_‐2 were further analyzed using the X‐ray photoelectron spectroscopy (XPS) technique. The full scan XPS spectrum of the CuVO_x_‐2, as shown in Figure S2 (Supporting Information), contains three diverse peaks at 516.61, 529.48, and 934.03 eV, which belong to V 2p, O 1s, and Cu 2p, respectively. Figure 2c shows the Cu 2p XPS spectrum of CuVO_x_‐2 sample. The peaks located at 931.92 and 951.11 eV correspond to the Cu 2p_3/2_ and Cu 2p_1/2_, respectively, which confirms the presence of Cu^+^ in the CuVO_x_‐2.^[^ ^27^ ^]^ Moreover, the peaks for Cu 2p_3/2_ and Cu 2p_1/2_ located at 934.39 and 953.60 eV, respectively indicate the existence of Cu^2+^. The satellite peak is also positioned at 941.66 eV.^[^ ^28^ ^]^ The V presence of CuVO_x_‐2 involved in the oxidation state of V^5+^, derived from the deconvoluted peaks at 516.54 eV (V 2p_3/2_), and 524.09 eV (V 2p_1/2_), respectively, as shown in Figure 2d.^[^ ^25^, ^29^ ^]^ The dominant presence of Cu^2+^ and V^5+^ in the CuVO_x_‐2 aligns with the Cu and V valence states observed in Cu_2_V_2_O_7_ and Cu_3_V_2_O_8_ crystals. To verify the porous structure of the as‐prepared samples, Brunauer Emmett–Teller (BET) analysis was performed on CuVO_x_‐1, CuVO_x_‐2, and CuVO_x_‐4. As shown in Figure 2e, all the samples exhibited steep H1 hysteresis loops in the pressure range of 0.9–0.96, indicating a narrow pore size distribution in the CuVO_x_ materials. The specific surface area values of the CuVO_x_‐1, CuVO_x_‐2, and CuVO_x_‐4 were determined to be 11.10, 17.29, and 16.58 m^2^ g^−1^, respectively. Remarkably, the observed surface area can be attributed to the occupation of pores within the Cu‐MOF by introduced vanadium species and their secondary nanoparticles. Furthermore, pore distribution results (Figure 2f) indicate the presence of numerous mesopores with a distribution centered below 5 nm, which persists during the conversion process. This coexistence of a mesoporous structure in the synthesized CuVO_x_ is advantageous for facilitating Zn^2+^ intercalation/deintercalation between the electrode and electrolyte.

**Figure 3:**
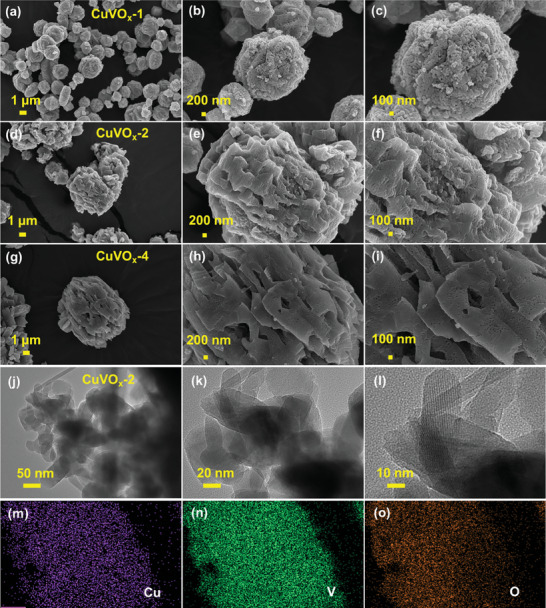
Low‐ and high‐magnification FE‐SEM images of the a–c) CuVO_x_‐1, d–f) CuVO_x_‐2, and g–i) CuVO_x_‐4 materials. j,k) Low‐ and high‐magnification TEM images and l) high‐resolution TEM image of the CuVO_x_‐2 material. m–o) EDS elemental mapping images of Cu, V, and O for the CuVO_x_‐2 material.

The morphologies and structures of the as‐prepared CuVO_x_ materials were characterized by field‐emission scanning electron microscope (FE‐SEM) analysis. **Figure** **3**a–i shows morphological transformations of CuVO_x_ at different concentrations of Cu‐MOF and V sources. Figure 3a–c shows the FE‐SEM images of CuVO_x_‐1, with minimal agglomeration and particles dispersed evenly throughout the matrix. As the concentration increases (CuVO_x_‐2), pronounced agglomeration occurs, leading to the formation of distinct clusters or aggregates of particles (Figure 3d–f). Remarkably, the CuVO_x_‐2 samples exhibited a balanced agglomerated structure, suggesting an optimal composition for achieving desirable material properties. This intermediate concentration allows for the formation of well‐defined clusters while still maintaining some level of particle dispersion. Furthermore, as the concentration increased (CuVO_x_‐4), extensive agglomeration became increasingly apparent, dominated by larger clusters, as shown in Figure 3g–i. The material's sensitivity to changes in the V source concentration underscores the critical need for precise composition control in shaping material properties for energy storage applications. In addition, transmission electron microscope (TEM) images of the material CuVO_x_‐2 displayed distinct clusters with interconnected void spaces, indicating a highly porous structure (Figure 3j–l). This porous morphology offers a large surface area for Zn^2+^ adsorption and promotes effective ion diffusion, thereby boosting the material's electrochemical properties. The energy‐dispersive X‐ray spectroscopy (EDS) elemental mapping results clearly demonstrated that the distribution of V, Cu, and O elements in the CuVO_x_‐2 sample was homogeneous (Figure 3m–o), which is in good agreement with the results of XPS.

### 2.2 Electrochemical Measurements of the Fabricated CuVO_x_ Cathode Materials

**Figure 4:**
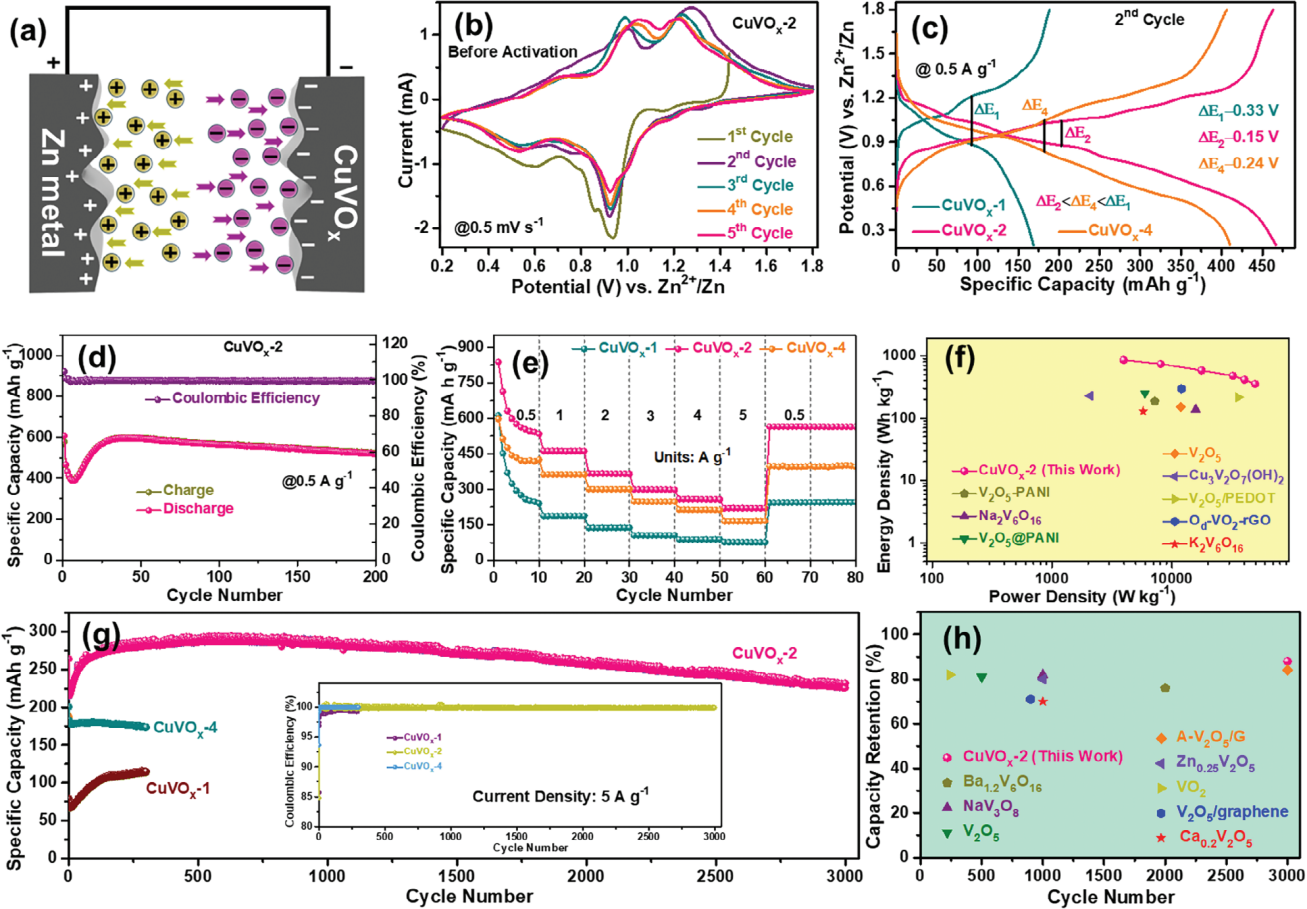
a) Schematic of the AZIB based on CuVO_x_ cathode and Zn metal. b) CV curves of the CuVO_x_‐2 electrode at 0.5 mV s^−1^. c) GCD profiles of the CuVO_x_ electrodes at the 2^nd^ cycle. d) Charge–discharge cycling performance of the CuVO_x_‐2 electrode at 0.5 A g^−1^ over 200 cycles. e) Rate performance at the current densities ranging from 0.5 to 5 A g^−1^. f) Ragone plots for the CuVO_x_‐2 electrode and other reported cathodes. g) Long‐term cycling performance of the CuVO_x_ electrodes at 5 A g^−1^. h) Comparison of capacity retention vs cycle number with other V‐based cathodes.

To assess the electrochemical performance of the CuVO_x_ electrodes, CR2032 type coin‐cells were constructed, featuring Cu‐MOF, CuVO_x_‐1, CuVO_x_‐2, and CuVO_x_‐4 cathodes, paired with a Zn metal anode and a 3 m zinc trifluoromethanesulfonate (ZnC_2_F_6_O_6_S_2_) aqueous electrolyte. This setup facilitates a comprehensive examination of their behavior within the battery system (**Figure** **4**a). Prior to electrochemical property measurements on the prepared electrodes, it is essential to coat the as‐prepared material onto the carbon cloth (CC) substrate, as detailed in Section S1.3 (Supporting Information). The specific capacity of all the fabricated AZIBs utilizing these prepared cathodes was then calculated based on the active material coated onto the CC. The comparative analysis presented in Figures S3–S6 (Supporting Information), along with Figure 4, collectively revealed the superior electrochemical performance of the CuVO_x_‐2 electrode in contrast to the CuVO_x_‐1 and CuVO_x_‐4 electrodes. Specifically, Figure 4b and Figure S3a,b (Supporting Information) show the initial five cyclic voltammetry (CV) curves for CuVO_x_‐2, CuVO_x_‐1, and CuVO_x_‐4, respectively within the potential window range of 0.2–1.8 V versus Zn^2+^/Zn, conducted at a scan rate of 0.5 mV s^−1^. In the initial cycle, all the electrodes demonstrated three distinct redox processes, indicative of an electrochemical activation phase. Notably, significant redox peaks emerged at ≈1.22 and 0.92 V during the initial CV curves. It is noteworthy that the reduction peaks observed in the initial CV curves manifest slight variations compared to subsequent cycles, likely stemming from the displacement phenomenon of Zn^2+^/Cu^2+^ during the initial Zn^2+^ insertion/de‐insertion process within the CuVO_x_ electrode matrix. Subsequent CV analyses displayed three primary pairs of redox peaks occurring at 1.27 V/1.18 V, 1.00 V/0.92 V, and 0.69 V/0.59 V, attributed to the intricate Zn^2+^ insertion/de‐insertion dynamics in the CuVO_x_‐2 electrode.^[^ ^30^ ^]^ Figure S5 (Supporting Information) shows the galvanostatic charge–discharge (GCD) cycling performance of the CuVO_x_‐1, CuVO_x_‐2, and CuVO_x_‐4 electrodes at 0.5 A g^−1^. While the GCD profiles do not directly indicate major irreversible phase transitions for the CuVO_x_‐2 electrode, the detailed analysis revealed that the CuVO_x_‐2 electrode demonstrates significantly better cycling stability and reversibility than those of the CuVO_x_‐1 and CuVO_x_‐4 electrodes. Specifically, the CuVO_x_‐2 electrode achieved a 2^nd^ cycle capacity of 466 mAh g^−1^ with a high Coulombic efficiency (≈99%), which is improved to 100% after 16 cycles, suggesting the enhanced reversibility of Zn‐ion insertion/extraction with cycling. This increased reversibility aligns with the concept of electrochemical activation, where Zn‐ion storage stabilizes as the electrode's structure adapts, leading to greater cycling stability. Further evidence from the CV profiles (Figure S4, Supporting Information) showed reduced polarization in the CuVO_x_‐2 electrode, indicating more efficient Zn‐ion transport over multiple cycles. In contrast, the CuVO_x_‐1 and CuVO_x_‐4 electrodes exhibited lower Coulombic efficiency, greater irreversibility, and broader CV peaks with larger potential gaps between oxidation and reduction, suggesting poorer Zn‐ion insertion/extraction and possible structural degradation. The superior cycling performance of the CuVO_x_‐2 electrode can be attributed to its optimized electrochemical properties, making it more suitable for long‐term Zn‐ion storage compared to the CuVO_x_‐1 and CuVO_x_‐4 electrodes which suffer from structural instability and poor conductivity. In Figure 4c, the GCD curves for the CuVO_x_ electrodes during the 2^nd^ cycle, measured at a scan rate of 0.5 mV s^−1^, revealed key insights into the performance of each electrode. The CuVO_x_‐2 electrode displayed a distinctly lower polarization potential (∆E_2_<∆E_4_<∆E_1_) compared to the CuVO_x_‐1 and CuVO_x_‐4 electrodes. This reduction in polarization potential indicates improved charge/discharge kinetics and reduced energy losses, reflecting the enhanced efficiency in the electrochemical processes.^[^ ^31^, ^32^, ^33^ ^]^ The CuVO_x_‐2 electrode's superior specific capacity of 466 mAh g^−1^, as compared to 168 mAh g^−1^ for the CuVO_x_‐1 electrode and 410 mAh g^−1^ for the CuVO_x_‐4 electrode, demonstrates that capacity is influenced by additional factors beyond polarization. Specifically, the higher capacity of CuVO_x_‐2 can be attributed to its optimized structural properties and better ion diffusion characteristics which facilitate more effective Zn‐ion storage and transport. Thus, while a lower polarization potential contributes to improved efficiency, the higher capacity of the CuVO_x_‐2 electrode is also due to its favorable structural and electrochemical attributes. These findings underscore the importance of considering both kinetic performance and material characteristics in evaluating the overall efficacy of Zn‐ion storage materials.

The investigation into the cycling performance of CuVO_x_ cathodes at 0.5 A g^−1^ is depicted in Figure 4d along with Figure S6a,b (Supporting Information). Initially, the CuVO_x_‐1, CuVO_x_‐2, and CuVO_x_‐4 electrodes displayed the specific capacities of 322, 607, and 490 mAh g^−1^, respectively, which experienced a significant decrease at the 2^nd^ cycle. This phenomenon can be elucidated by the presence of Zn ions introduced at “dead Zn^2+^ sites”, which resists effective removal from the CuVO_x_ lattice during subsequent charging cycles.^[^ ^34^, ^35^, ^36^ ^]^ Moreover, the specific capacity of the CuVO_x_‐1, CuVO_x_‐2, and CuVO_x_‐4 electrodes exhibited a gradual increase from 168, 466, and 410 mAh g^−1^ to 268, 591, and 639 mAh g^−1^ after 60, 33, and 25 cycles, respectively, attributed to the electrochemical activation facilitated by Zn^2+^ insertion/extraction. Furthermore, with the progression of cycling, there was a notable augmentation in the permeation of the aqueous electrolyte into the electrode material, leading to the enhanced utilization of active material.^[^ ^37^ ^]^ This phenomenon resulted in the expansion of interlayer spacing and activation of the electrode, consequently culminating in an augmented specific capacity. Of particular significance is the consistent outperformance of the CuVO_x_‐2 electrode throughout the cycling regime, which exhibited a specific capacity of 519 mAh g^−1^, surpassing those of the CuVO_x_‐1 (246 mAh g^−1^) and CuVO_x_‐4 (405 mAh g^−1^) electrodes. Furthermore, even after 200 cycles, the capacity retention rate of the CuVO_x_‐2 electrode remained commendably high at 86%, underscoring the efficacy of introducing an optimal ratio of Cu:V ions in enhancing Zn^2+^ storage performance. Moreover, the Coulombic efficiency of the CuVO_x_‐2 electrode maintained proximity to 100% across the entirety of the cycling process, accentuating its exceptional electrochemical reversibility and reliability.

The rate performance of batteries holds significant importance for practical applications. Figure 4e illustrates the rate performance upon cycling at different current densities. The AZIBs underwent 10 cycles at each current density, in which the CuVO_x_‐2 cathode displayed the reversible capacities of 534, 461, 365, 298, 257, and 220 mAh g^−1^ at the current densities ranging from 0.5 to 5 A g^−1^. These values surpassed those of the CuVO_x_‐1 (243, 187, 137, 105, 88, and 76 mAh g^−1^) and CuVO_x_‐4 (425, 362, 300, 247, 212, and 166 mAh g^−1^) cathodes at identical current densities, respectively. Notably, when the current density reverted to 0.5 A g^−1^, the CuVO_x_‐2 electrode resumed its initial capacity of 564 mAh g^−1^, indicating the favorable kinetics of Zn^2+^ insertion/extraction reactions. Additionally, the CuVO_x_‐2 electrode maintained ≈100% Coulombic efficiency across various rate densities, surpassing the CuVO_x_‐1 and CuVO_x_‐4 electrodes. This superior rate performance of the CuVO_x_‐2 cathode exceeded that of the reported V‐based cathodes in AZIBs. Furthermore, Figure S7 (Supporting Information) depicts the Ragone plots of the CuVO_x_‐1, CuVO_x_‐2, and CuVO_x_‐4 cathodes for AZIBs. The CuVO_x_‐2 cathode exhibited superior energy and power densities over the CuVO_x_‐1 and CuVO_x_‐4 cathodes (a higher energy density of 0.85 kWh kg^−1^ at 0.5 A g^−1^ and power density of 49.63 kW kg^−1^ at 5 A g^−1^). Moreover, the superior energy and power densities of the CuVO_x_‐2 cathode were compared with the previously reported AZIBs,^[^ ^38^, ^39^, ^40^, ^41^, ^42^, ^43^, ^44^, ^45^ ^]^ as demonstrated in Figure 4f. During continuous cycling at 5 A g^−1^, the CuVO_x_‐2 cathode exhibited remarkable stability, with only a slight decline in capacity observed during the initial cycles. Even after 3000 cycles, the CuVO_x_‐2 cathode maintained an impressive capacity retention of ≈88%, as depicted in Figure 4g. In contrast, the CuVO_x_‐1 and CuVO_x_‐4 cathodes displayed lower capacity retention, reaching the values of ≈82% and ≈86%, respectively after 300 cycles. The intermediate V content in the CuVO_x_‐2 electrode significantly facilitates electron transfer, resulting in a much higher capacity compared to the low V content in the CuVO_x_‐1 electrode. Despite the CuVO_x_‐4 with a higher V content, it struggles to achieve its full capacity potential due to structural agglomeration issues and a less specific surface area. Notably, the CuVO_x_‐2 presented a distinct cluster‐type NP configuration, which may enhance interparticle spacing but also raises concerns about structural instability during the continuous Zn^2+^ insertion/extraction process, potentially affecting cycling performance. However, the 1D arrangement of the stacked NPs in the CuVO_x_‐2 not only increases surface reactive sites but also facilitates Zn ion transportation, contributing to its enhanced structural stability. Furthermore, as shown in Figure 4h, the CuVO_x_‐2 electrode exhibited superior cycling life and capacity retention relative to many other cathode materials.^[^ ^46^, ^47^, ^48^, ^49^, ^50^, ^51^, ^52^, ^53^ ^]^ This enhanced performance is indicative of the CuVO_x_‐2 electrode's stability and effectiveness over extended charge/discharge cycles. The comprehensive comparison of cycling performance for various V‐based materials is detailed in Table S1 (Supporting Information). This table highlights that the CuVO_x_‐2 electrode outperforms other cathode materials in terms of longevity and capacity maintenance, emphasizing its potential for high‐performance long‐lasting energy storage applications. Furthermore, to emphasize the practical application of the CuVO_x_‐2 cathode, we designed a pouch cell by sandwiching a separator and 3 m aqueous Zn trifluoromethanesulfonate electrolyte between the CuVO_x_‐2 cathode and Zn anode (Figure S8, Supporting Information). This pouch cell configuration was specifically chosen for its significance in real‐world applications due to its compact design, scalability, and ease of assembly. To evaluate the practical viability of the Zn//CuVO_x_‐2 pouch cell, two pouch cells were connected in series and charged to provide sufficient energy to power a stopwatch and an electric motor fan (as shown in Figure S8b–e, Supporting Information). The ability to power these external devices provides solid evidence of the Zn//CuVO_x_‐2 pouch cell's real‐time operational capability. This experimental demonstration highlights the robust performance of the Zn//CuVO_x_‐2 battery system, showing its potential in practical energy storage devices. By successfully driving common electronic devices, the pouch cell confirmed its suitability for applications in portable electronics and small‐scale power systems. Moreover, this result showcases the versatility and reliability of the CuVO_x_‐2 cathode material, suggesting that it can be adapted to a wide range of portable and stationary energy storage systems. This flexibility is crucial for the ongoing development of sustainable energy technologies, offering significant potential for further optimization and scaling. Enhancements to the energy density, cycle life, and efficiency of the Zn//CuVO_x_‐2 pouch cell could position this material as a key player in future energy storage solutions, contributing to the advancement of green energy technologies and reducing reliance on traditional battery systems.

### 2.3 Electrochemical Reaction Kinetics

**Figure 5:**
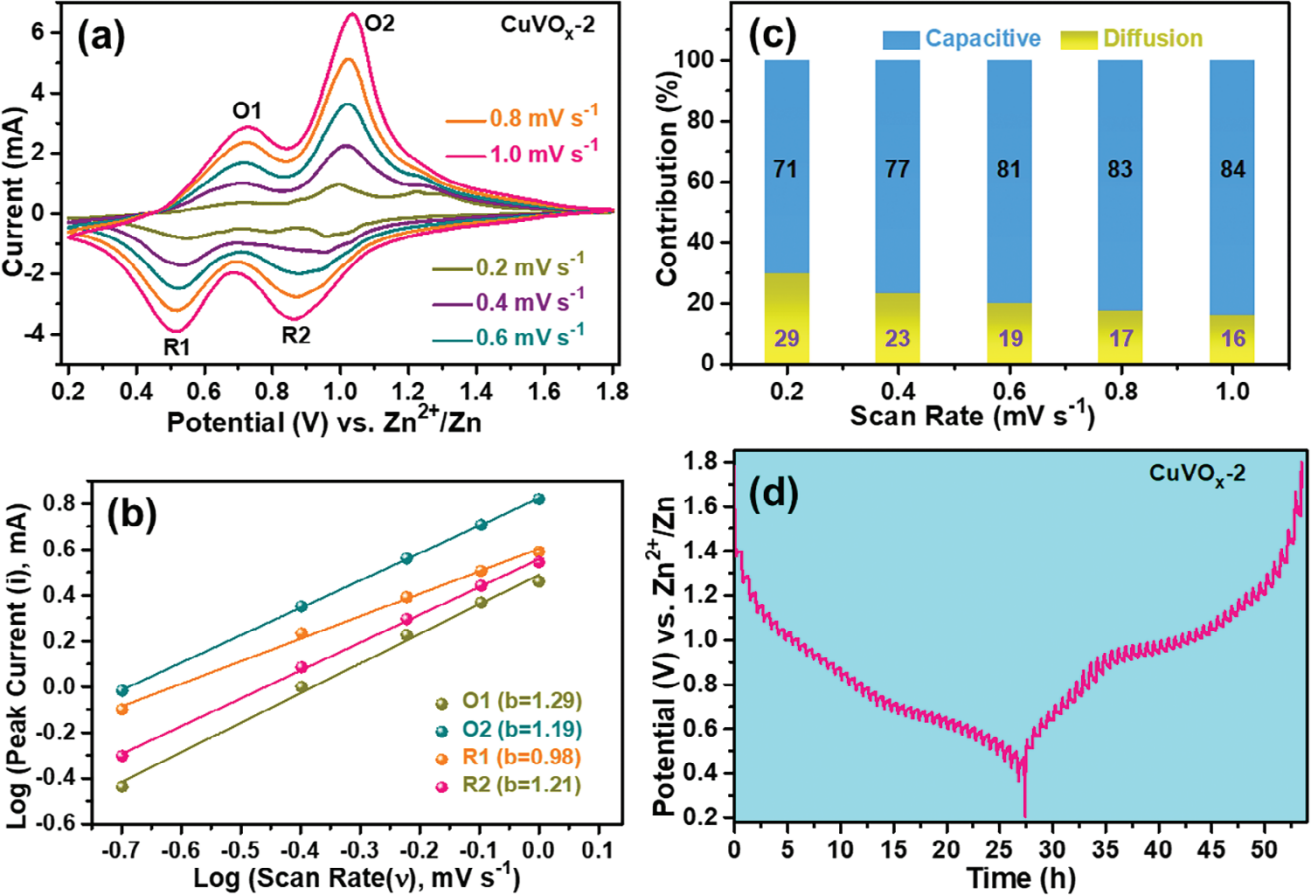
a) CV curves under different scan rates, b) corresponding relation of log(i) vs log(ν), c) capacitve and diffusion‐controlled contribution at various scan rates, and d) GITT curve at the 2^nd^ cycle of the CuVO_x_‐2 cathode.

The underlying mechanism for the superior electrochemical performance of CuVO_x_‐2 was investigated by electrochemical impedance spectroscopy (EIS), CV, and galvanostatic intermittent titration technique (GITT) measurements. To evaluate the capacitive and diffusion‐controlled contributions to the capacity of the CuVO_x_‐2 electrode, the CV analysis was conducted at various scan rates ranging from 0.2 to 1.0 mV s^−1^ (**Figure** **5**a). The CV curves of the CuVO_x_‐2 electrode displayed consistent shapes at different scan rates, suggesting a strong ability to accommodate Zn^2+^ intercalation and deintercalation. Initially, multiple pairs of redox peaks were evident in the CV test conducted at a scan rate of 0.5 mV s^−1^ (Figure 4b). However, as the number of scans and scan rate increased, these additional peaks gradually disappeared, leaving behind two distinct pairs of redox peaks. This evolution implies that the cathode material underwent a brief activation process during the CV test before achieving stability. The scan data analysis revealed that the current (i) of CV curves could be described by a power‐law formula with the scan rate (ν) according to the formula below:^[^ ^8^ ^]^

(1) $$i=av^{b}$$

In the above equation, variables a and b are subject to change, while i represents the peak current at the scan rate. The b‐value can be determined by linearly fitting the slope of log(i) versus log(ν). Typically, b‐values are located in the range of 0.5–1.0. When the b‐value is 0.5, it suggests a diffusion‐controlled process, whereas the b‐value of 1 indicates a capacitive process. From the redox peaks demonstrated in Figure 5b, the b‐values are close to 1, suggesting a capacitive process. The calculated results indicate that the CuVO_x_‐2 electrode primarily exhibits capacitor‐like kinetics, likely attributed to surface pseudocapacitance. The predominance of capacitive contribution reflects rapid Zn ion storage behavior, resulting in exceptional rate capability. Moreover, the Zn ion storage process can be divided into capacitive (k_1_ν) and diffusion‐controlled (k_2_ν^0.5^) processes at a specific scan rate, as demonstrated below:^[^ ^54^ ^]^

(2) $$i=k_{1}v+k_{2}v^{0.5}$$

The provided CV curves illustrate both surface and diffusion‐controlled responses. Through calculations, it is determined that the current attributed to the capacitive response accounts for ≈81% at a scan rate of 0.6 mV s^−1^, indicating favorable high‐rate performance. Figure 5c depicts the division of capacity into capacitive and diffusion‐controlled parts. Notably, the ratio of capacitive contribution to the total capacity steadily increases from 71% to 84% with increasing scan rates, implying the rapid and highly reversible reaction kinetics at higher scan rates. The electrical conductivity and Zn ion diffusion capabilities of the CuVO_x_ cathodes were assessed using EIS measurements (Figure S9, Supporting Information). In the Nyquist plots of the three cathodes, a semicircle appears in the high‐frequency region, representing the charge‐transfer resistance (R_ct_), while a diagonal line is observed in the low‐frequency region, indicative of ion diffusion resistance. Notably, the CuVO_x_‐2 cathode exhibited a significantly lower R_ct_ value of 264.9 Ω compared to the CuVO_x_‐1 (434.01 Ω) and CuVO_x_‐4 (395.6 Ω) cathodes, indicating enhanced conductivity. Additionally, the slope of the diagonal line for the CuVO_x_‐2 cathode suggests faster Zn^2+^ diffusion velocity and improved ion storage kinetics when compared to the CuVO_x_‐1 and CuVO_x_‐4 cathodes.^[^ ^55^ ^]^ The results from the GITT test provide further evidence in support of this conclusion. The GITT curves of the CuVO_x_‐2, CuVO_x_‐1, and CuVO_x_‐4 cathodes are depicted in Figure 5d and Figure S10 (Supporting Information), respectively. During the test, the system underwent a pulse of 0.05 A g^−1^ for 10 min following a rest period of 30 min. The Zn ion diffusion coefficient (D_Zn_^2+^) can be calculated using the equation below:^[^ ^56^ ^]^

(3) $$D_{Zn^{2+}}=\frac{4}{\pi \tau }{\left(\frac{m_{B}V_{M}}{M_{B}S}\right)}^{2}{\left(\frac{\Delta E_{s}}{\Delta E_{\tau }}\right)}^{2},\left(\tau <<L^{2}/D\right)$$

where D_Zn_^2+^ represents the ion diffusion coefficient (cm^2^ s^−1^), τ denotes the constant current pulse time (s), m_B_ signifies the quantity of the active material (g), V_M_ represents the molar volume of the active material (cm^3^ mol^−1^), and M_B_ indicates the relative molecular mass (g mol^−1^) of the active material. S refers to the area where the electrode is in contact with the electrolyte (cm^2^), ∆E_s_ represents the steady‐state voltage change induced by the current pulse, and ∆E_τ_ denotes the potential change (V) during the constant current pulse. The D_Zn_^2+^ values of the CuVO_x_‐2 electrode are in the range of 10^−10^ to 10^−11^ cm^2^ s^−^^1^ during the discharge and charge processes (Figure S11b, Supporting Information), exceeding the values obtained on the CuVO_x_‐1 (Figure S11a, Supporting Information) and CuVO_x_‐4 (Figure S11c, Supporting Information) electrodes. The result directly reflects the rapid transport of Zn ions in the CuVO_x_‐2 electrode. Another evidence to prove the rapid electrochemical kinetics of the CuVO_x_‐2 electrode can be found by calculating the relaxation time constant (τ_0_). The τ_0_ value is relevant to the frequency (f_max_) commensurate with the maximum imaginary impedance (Z”) of the semicircle in the EIS results. The constant can be calculated using the following formula:

(4) $$\tau =\frac{1}{2\pi f_{\max}}$$

Accordingly, the τ_0_ values were calculated to be 0.16, 0.06, and 0.10 s for the CuVO_x_‐1, CuVO_x_‐2, and CuVO_x_‐4 cathodes, respectively. The lower τ_0_ value for the CuVO_x_‐2 cathode demonstrates its faster kinetics behavior. Based on the EIS, GITT, and CV results discussed above, it can be concluded that the improvement in electrochemical performance can be achieved by optimizing the contraction ratio of Cu:V in the CuVO_x_ material. This optimization enhances conduction, promotes Zn ion diffusion dynamics, and increases pseudocapacitive contribution. Such a strategy effectively enhances the overall electrochemical behavior of the CuVO_x_ material, thereby highlighting its potential for advanced energy storage applications.

### 2.4 Electrochemical Mechanism

**Figure 6:**
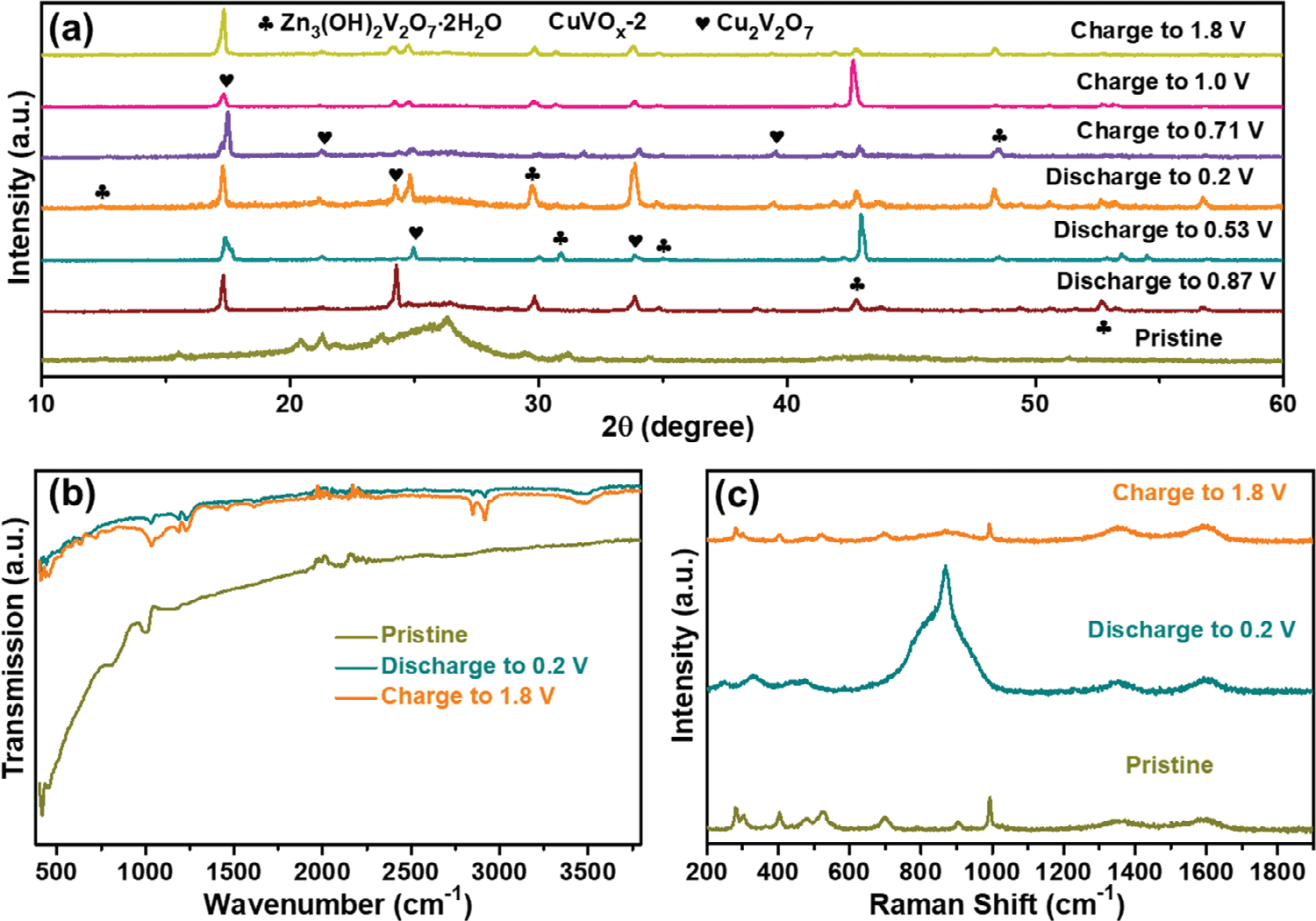
Zn^2+^ insertion/extraction process and mechanism of the CuVO_x_‐2 cathode. a) Ex situ XRD patterns, b) FT‐IR spectra, and c) Raman spectra at the selected charge–discharge states.

The structural transformation and electrochemical reaction kinetics of the CuVO_x_‐2 electrode were studied using ex situ XRD, Fourier‐transmission infrared (FT‐IR), and Raman characterizations. **Figure** **6**a shows the ex‐situ XRD patterns of the CuVO_x_‐2 electrode material at various discharge/charge potential states. During the discharge process, Zn^2+^ is inserted into the interlayer of the CuVO_x_‐2 electrode material, causing shifts in the diffraction peaks at ≈17.30° and 21.32° toward lower 2θ degree values due to a slight expansion of the interlayer spacing of V─O. However, the peaks at 24.26° and 33.8° initially exhibited a positive shift, followed by a negative shift during the discharge process. This shrinkage is possibly attributed to the enhanced electrostatic interaction between the inserted Zn^2+^ and V─O layers.^[^ ^46^, ^57^ ^]^ After the discharge process, the peaks shifted toward higher 2θ degree values during the charging process, before returning to lower 2θ° values during subsequent charging. This indicates a reversible process of Zn^2+^ insertion/extraction. Notably, a new phase emerged at 12.3°, 29.8°, 30.1, 34.2, 43.1°, 48.4°, and 52.7° when discharged to 0.53 V, which matches the characteristics of Zn_3_(OH)_2_V_2_O_7_⋅nH_2_O (#50‐0570).^[^ ^58^ ^]^ This suggests that Zn_3_(OH)_2_V_2_O_7_⋅nH_2_O appears on the electrode surface during discharge, as observed in other V‐based cathodes. This means a propensity to form on the electrode surface due to the enhanced interlaminar binding energy resulting from Zn^2+^ ingress into the oxide layers.^[^ ^59^ ^]^ The newly formed phase disappeared during the subsequent charging process, indicating that the reversible phase transition of Zn_3_(OH)_2_V_2_O_7_·nH_2_O occurred during the discharge process. The ex situ FT‐IR spectra in Figure 6b show no significant shift in the characteristic peaks under selected charge–discharge states, implying the stability of these components during cycling, which is beneficial for long‐term performance.^[^ ^60^ ^]^ It is important to note that the peak at 3472.5 cm^−1^ represents the O─H stretching vibration, which becomes weaker during charging and stronger during discharging and remains consistent with the bending vibration O─H change of 1639.2 cm^−1^. The Raman spectra of the CuVO_x_‐2 electrode in different charge–discharge states are shown in Figure 6c. Several Raman bands were observed at specific frequencies, including 280.9, 302.5, 402.9, 526.5, 699.4, 903.2, and 992.7 cm^−1^. The band at 280.9 cm^−1^ is associated with the channel‐structured copper vanadate, while the band at 699.4 cm^−1^ can be attributed to the V─O─V asymmetric vibration. The Raman frequencies at 903.2 cm^−1^ correspond to the stretching vibration of V─O bonds and terminal V─O─Cu or V─O─V vibrations.^[^ ^61^ ^]^ However, the Raman bands at 402.9 and 992.7 cm^−1^ may be related to signals from the binder and conductive agent. During discharging from pristine to 0.2 V, the Raman band at 280.9 cm^−1^ becomes weaker, while the band at 903.2 cm^−1^ becomes stronger and broader. This might indicate the insertion of Zn^2+^ accompanied by copper oxidation from Cu^+^ to Cu^2+^. Conversely, during charging from 0.2 to 1.8 V, the Raman band at 280.9 cm^−1^ becomes stronger and narrower, gradually returning to its original state at 903.2 cm^−1^, possibly due to the extraction of Zn^2+^ from the lattice.

## 3 Conclusion

In conclusion, we developed a series of CuVO_x_ materials in which V ions were integrated into the porous Cu‐MOF framework structure. The CuVO_x_‐1, containing low V content, showed minimal agglomeration, with particles evenly dispersed throughout the matrix. With an increase in V content in CuVO_x_, the pronounced agglomeration in CuVO_x_‐2 was observed, leading to the formation of distinct clusters or aggregates of particles and extensive agglomeration in the CuVO_x_‐4, dominated by larger clusters. As a result, the CuVO_x_‐2 electrode, used as the AZIB cathode, exhibited a higher specific capacity of 519 mAh g^−1^ at 0.5 A g^−1^, superior rate performance of 220 mAh g^−1^ at 5 A g^−1^, and capacity retention of 88% after 3000 cycles at 5 A g^−1^, outperforming the CuVO_x_‐1 and CuVO_x_‐4 electrodes. A series of ex situ characterizations to study the charge/discharge products demonstrated their highly stable structure and reversibility during the Zn^2+^ intercalation/deintercalation process. The excellent capability and ultra‐stability of the special porous agglomerated cluster particles suggested CuVO_x_ materials as a robust cathode for AZIBs. This work not only advances the current understanding of structure regulation in MOF‐derived porous materials but also identifies a promising candidate cathode for other rechargeable batteries, which paves the way for pioneering developments and improvements in energy storage technologies.

## 4 Experimental Section

All the chemicals and materials utilized in this experiment are detailedly outlined in the Supporting Information. The inorganic or organic chemicals are of analytical grade and were employed without additional purification steps. This ensures the integrity of the experimental process while maintaining simplicity and efficiency.

### Synthesis of Cu‐MOF

The synthesis of Cu‐MOF was carried out via a hydrothermal method. In a typical procedure, 2.22 g of copper nitrate was dissolved in 30 mL of deionized (DI) water, and 1.05 g of trimesic acid was dispersed in another 30 mL of DI water. These solutions were then transferred to a 100 mL capacity Teflon‐lined stainless steel autoclave, where heat treatment was conducted at 120 °C. After 12 h, the autoclave was allowed to naturally cool down to room temperature (RT). The resulting pale green‐colored Cu‐MOF precursor was obtained by centrifuging with DI water and ethanol several times, followed by drying at 145 °C for 12 h in an electric oven. The Cu‐MOF was further activated by immersing it in methanol for 48 h and subsequently dried at 145 °C.

### Synthesis of CuVO_x_ Nanostructures

For the synthesis of the CuVO_x_‐2 sample (with a Cu:V ratio of 1:2 by mass), a simple process was followed. Initially, 0.2 g (2 mmol) of ammonium metavanadate was dissolved in 40 mL of DI water containing 500 µL of ammonia solution (water‐based), and vigorously stirred for 0.5 h at 70 °C. Following this, 0.1 g of Cu‐MOF was uniformly dispersed in 40 mL of ethanol and gradually added to the above solution, stirring at RT for 2 h. Upon completion of the reaction, the solid product was retrieved via centrifugation, underwent thorough washing with DI water and ethanol multiple times, and dried in an electric oven at 80 °C overnight. Afterward, the dried sample underwent further thermal treatment at 400 °C for 2 h, with a gradual heating rate of 1 °C min^−1^ in a muffle furnace. Finally, the CuVO_x_‐2 NPs were collected after allowing the sample to cool down to ambient RT. Additionally, control samples with different mass ratios of ammonium metavanadate and Cu‐MOF (1:1 and 4:1, denoted as CuVO_x_‐1 and CuVO_x_‐4, respectively) were synthesized using a similar procedure as the CuVO_x_‐2.

Following the successful synthesis of CuVO_x_‐1, CuVO_x_‐2, and CuVO_x_‐4, the prepared samples underwent comprehensive physical characterizations and electrochemical measurements tailored for AZIBs. Detailed descriptions of all the physical characterizations and electrochemical measurements conducted in this experiment can be detailed in Sections S1.2 and S1.3 (Supporting Infomation).

## Conflict of Interest

The authors declare no conflict of interest.

## Supporting information



Supporting Information

## Data Availability

The data that support the findings of this study are available from the corresponding author upon reasonable request.
