# Supplementary material for: Unraveling Energy Storage Performance and Mechanism of Metal–Organic Framework‐Derived Copper Vanadium Oxides with Tunable Composition for Aqueous Zinc‐Ion Batteries
Source: Small Methods. 2024 Sep 17;9(1):2400819. doi: 10.1002/smtd.202400819 (PMC11740948; doi:10.1002/smtd.202400819)
Supplement: Supplementary file 1 — Supporting Information [file SMTD-9-2400819-s001.docx]

**Supporting Information**

**Unraveling Energy Storage Performance and Mechanism of Metal-Organic Framework-Derived Copper Vanadium Oxides with Tunable Composition for Aqueous Zinc-Ion Batteries**

Ashok Kumar Kakarla, Hari Bandi, Wasim Akram Syed, D. Narsimulu, R. Shanthappa, Jae Su Yu*

*Department of Electronics and Information Convergence Engineering, Institute for Wearable Convergence Electronics, Kyung Hee University, 1732 Deogyeong-Daero, Giheung-gu, Yongin-si, Gyeonggi-do, 17104, Republic of Korea*

*Corresponding author*: *jsyu@khu.ac.kr (J. S. Yu)*

**1. Experimental section**

**1.1. Chemicals**

All of the chemical reagents were directly used without any purging process. Trimesic acid (C_9_H_6_O_6_, 98%), zinc trifluoromethane sulfonate (Zn(CF_3_SO_3_)_2_, 98%), and copper nitrate (Cu(NO_3_)_2_·3H_2_O, ≥98%) were received from Sigma Aldrich Ltd., South Korea. Ammonium metavanadate (NH_4_VO_3_, 99%), polyvinylidene fluoride (PVDF, -(C_2_H_2_F_2_)_n_-), and N-methyl-2-pyrrolidone (NMP, C_5_H_9_NO) were obtained from Daejung Chemicals Ltd., South Korea. Zinc metal foil (Zn, Φ100 μm), CR-2032 coin cells, and super P carbon black were purchased from MTI Corp., South Korea. The carbon cloth (CC) was received from Nara Cell-Tech Corp., South Korea. The experiments were conducted using deionized (DI) water obtained from a Milli-Q water purification system (resistivity ~18 MΩ cm).

**1.2. Physical characterizations**

X-ray diffraction (XRD, D8 Advance (Bruker)) was conducted with Cu Kα (λ=1.5406 Å) at 40 kV and 40 mA in the 2θ range from 10° to 80° at a scanning rate of 3° min^-1^. The element content in the as-prepared materials was analyzed by inductively coupled plasma (ICP, Direct Reading Echelle ICP (LEEMAN)) spectroscopy. Field-emission scanning electron microscope (FE-SEM, LEO SUPRA 55, GENESIS 2000 (Carl Zeiss, EDAX) with an accelerated voltage of 15 kV) and transmission electron microscope (TEM, JEM-2100F (JEOL) with an operating voltage of 200 kV) were used to examine the surface morphology and substructure of the synthesized samples. The elemental mapping was recorded by energy-dispersive X-ray spectroscopy (EDS, Oxford INCA, Resolution 30 mm^2^ 136 eV at Mn Kα). Raman spectroscopy was studied with a 514 nm laser excitation source from Research grade Leica DM250 microscope. Fourier-transmission infrared (FT-IR, Spectrum One System (Perkin-Elmer)) spectroscopy analysis was carried out from 4000 to 400 cm^-1^. Thermo Electron KA 1066 Spectrometer with a monochromatic Al Kα source was used to perform X-ray photoelectron spectroscopy (XPS). N_2_ adsorption/desorption tests were performed to determine the Brunauer-Emmett-Teller (BET) specific surface area, pore volume, and pore size distribution (BELSORP-mini X).

**1.3. Electrochemical measurements**

The working electrode consisted of 70 wt% of the as-prepared samples (CuVO_x_), 10 wt% PVDF binder, and 20 wt% super P carbon in NMP solvent. The slurry was coated onto a CC substrate. After that, drying at 120 °C for 12 h was performed in an electric oven, and then, the substrate was cut into 12 mm circular disks. The mass loading was 0.9-1.6 mg cm^-2^. The cyclic voltammetry (CV) curves, galvanostatic charge-discharge (GCD) curves, and cycling performance with a potential range of 0.2-1.8 V vs. Zn^2+^/Zn were measured on the battery testing system (Wontech, WBCS3000) at room temperature. The galvanostatic intermittent titration technique (GITT) was performed under a modified GCD mode, in which an operation period includes two parts: a charge/discharge process lasting for 10 min at 0.1 A g^-1^ and a subsequent pause time for 30 min. The electrochemical impedance spectroscopy (EIS) plot was measured with an alternating voltage amplitude of 5 mV in a frequency range of 0.01 Hz to 100 kHz at an open-circuit potential. All the electrochemical measurements were conducted at room temperature (26 °C).


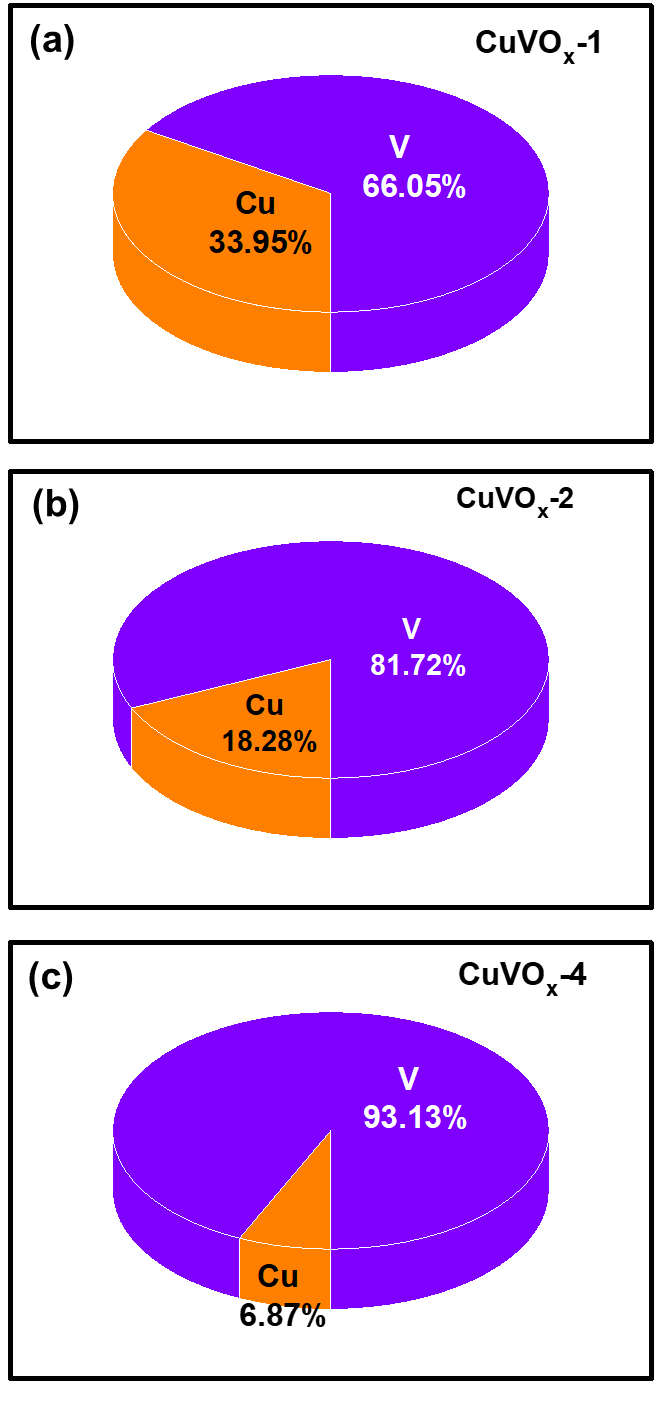


**Figure S1.** ICP spectroscopy elemental content of the (a) CuVO_x_-1, (b) CuVO_x_-2, and (c) CuVO_x_-4 materials.


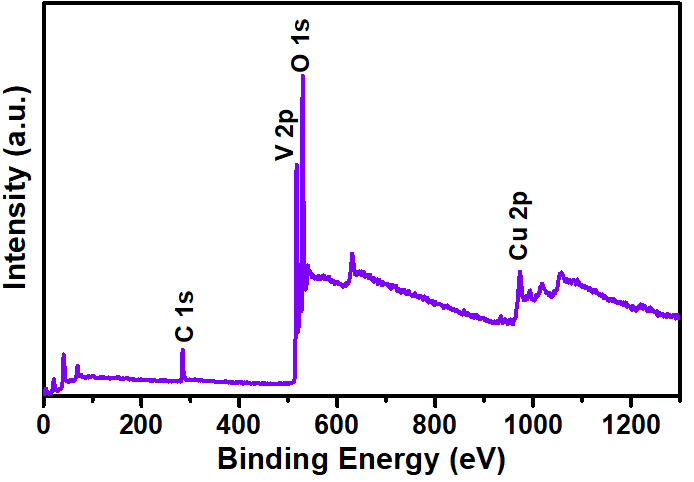


**Figure S2.** XPS survey scan spectrum of the CuVO_x_-2 material.


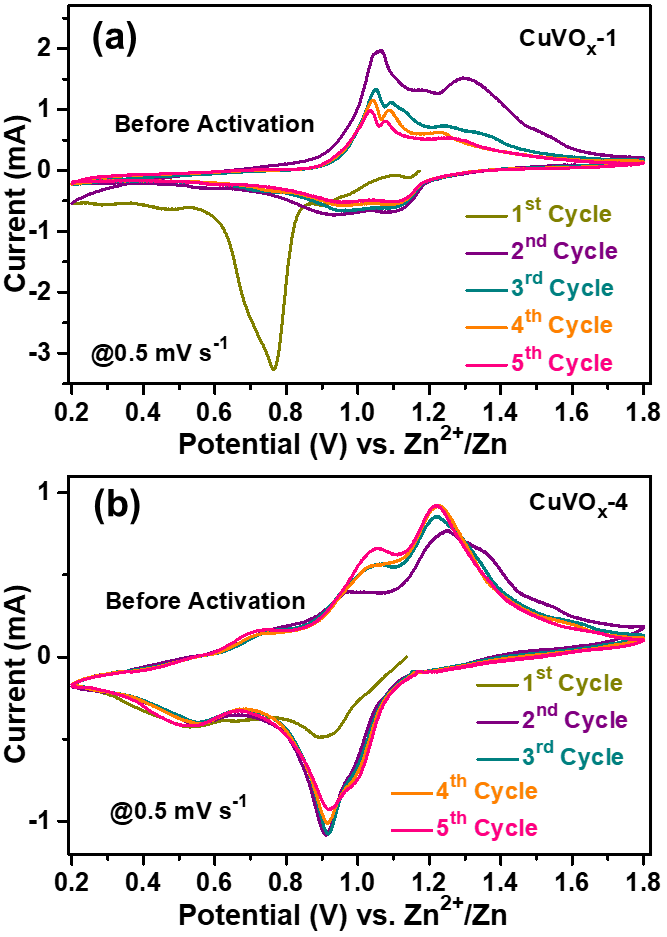


**Figure S3.** CV curves of the (a) CuVO_x_-1 and (b) CuVO_x_-4 electrodes at a scan rate of 0.5 mV s^-1^.


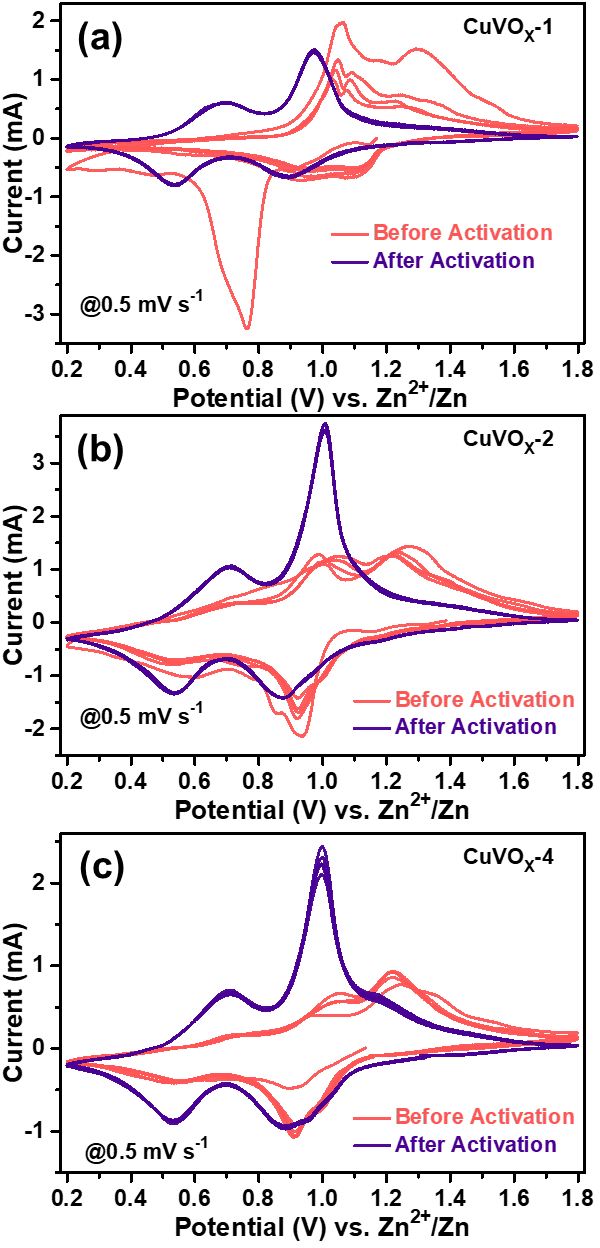


**Figure S4.** CV curves of the (a) CuVO_x_-1, (b) CuVO_x_-2, and (c) CuVO_x_-4 electrodes before and after activation states at a scan rate of 0.5 mV s^-1^.


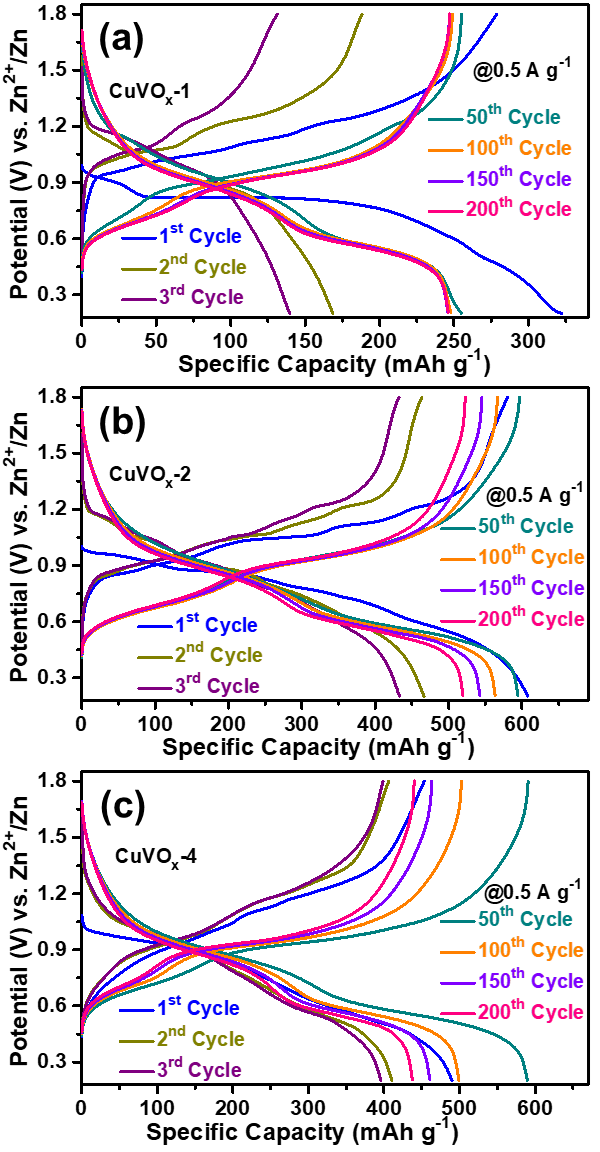


**Figure S5.** GCD plots of the (a) CuVO_x_-1, (b) CuVO_x_-2, and (c) CuVO_x_-4 electrodes at 0.5 A g^-1^.


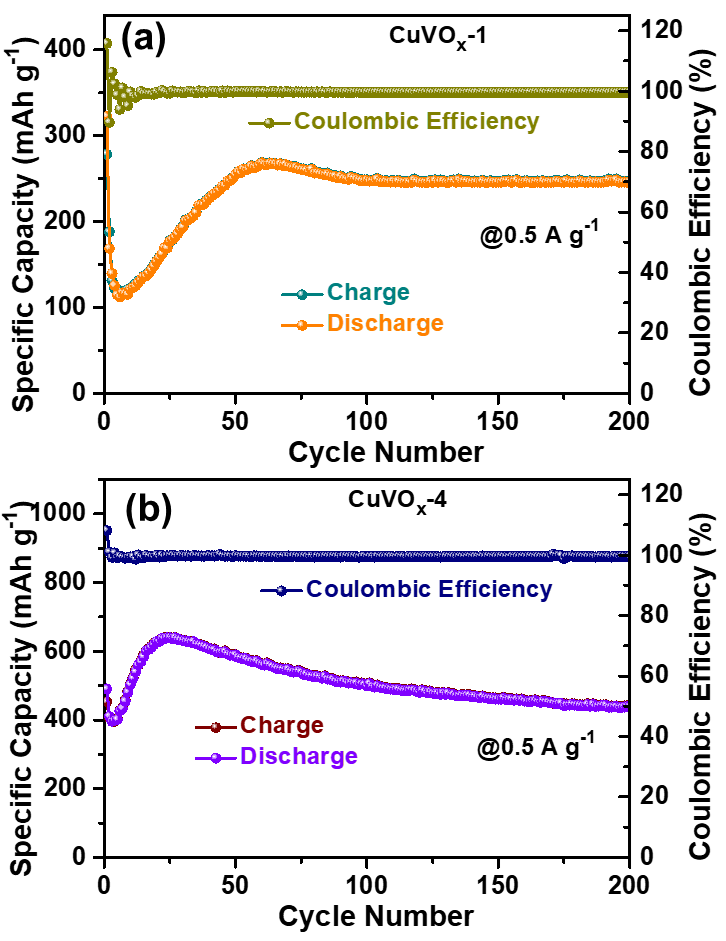


**Figure S6.** Charge-discharge cycling performance of the (a) CuVO_x_-1 and (b) CuVO_x_-4 electrodes at 0.5 A g^-1^ over 200 cycles.


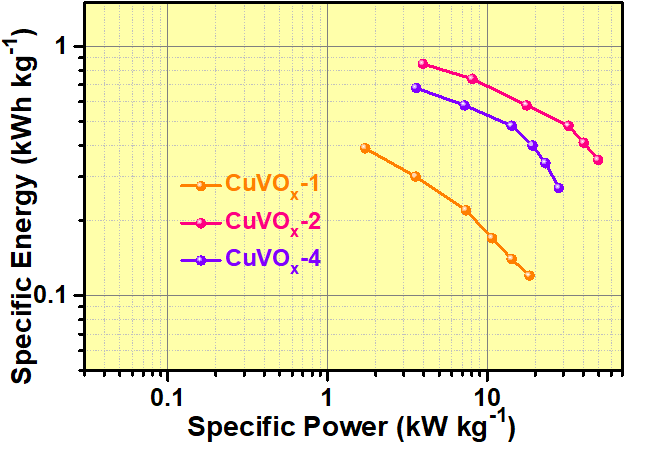


**Figure S7.** Comparative Ragone plots of energy and power densities for the as-fabricated CuVO_x_ electrodes.


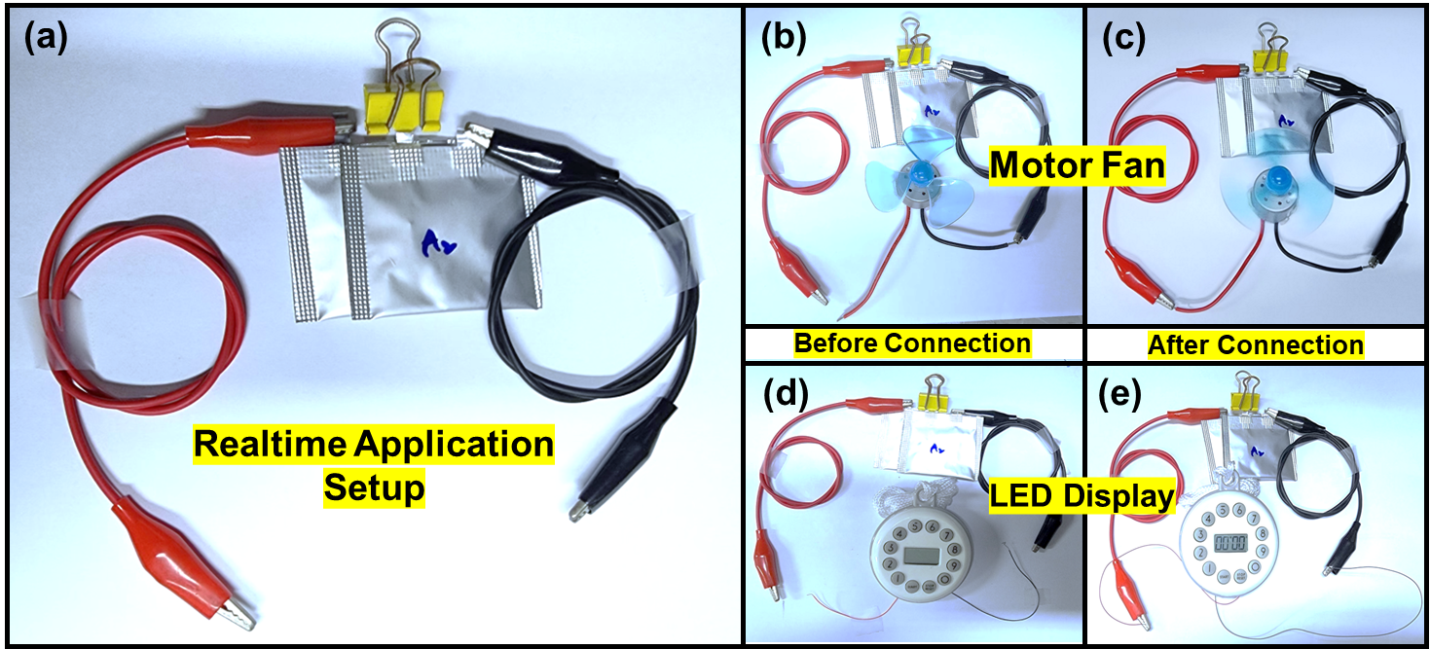


**Figure S8.** (a) Photographic images of serially connected Zn//CuVO_x_-2 pouch cell devices. Photographic images of the (b, c) stopwatch and (d, e) electronic motor fan before and after connections.


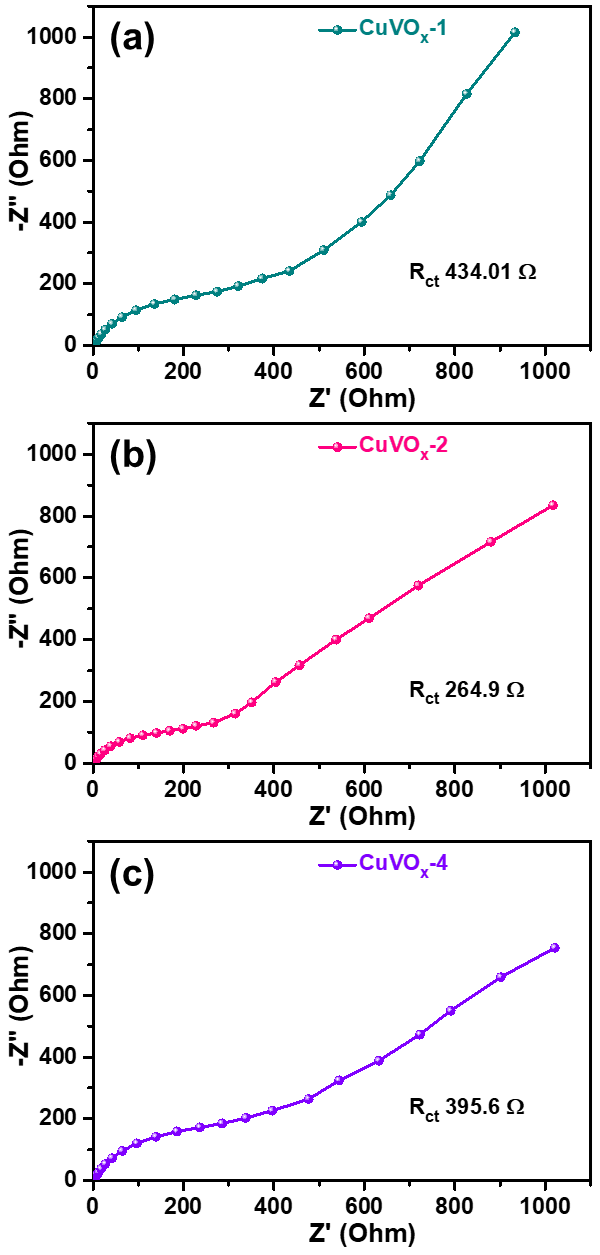


**Figure S9.** EIS plots of the (a) CuVO_x_-1, (b) CuVO_x_-2, and (c) CuVO_x_-4 electrodes.


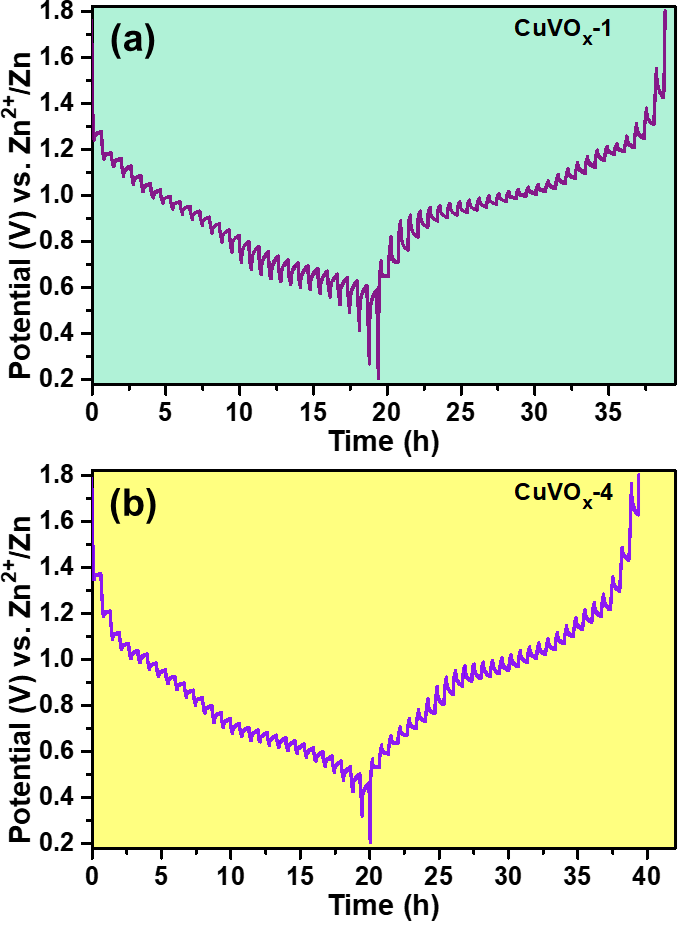


**Figure S10.** GITT plots of the (a) CuVO_x_-1 and (b) CuVO_x_-4 electrodes at the 2^nd^ cycle.


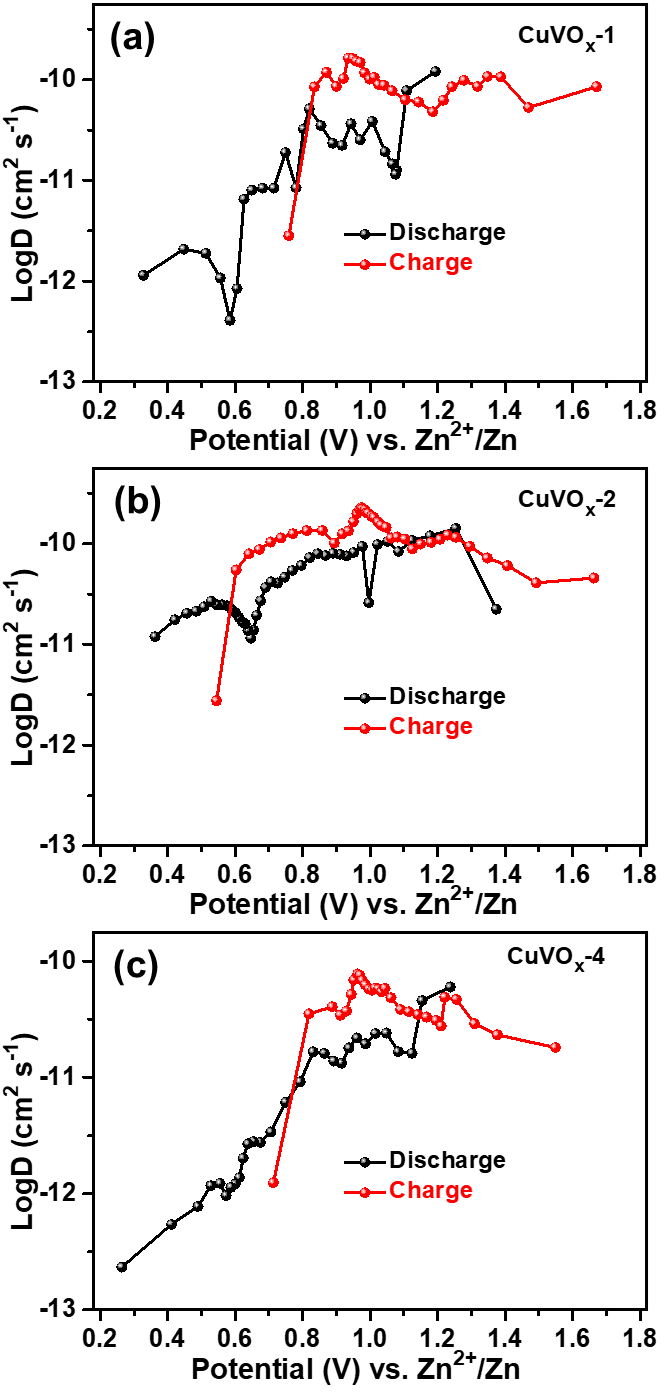


**Figure S11.** Calculated ionic diffusion coefficients of the (a) CuVO_x_-1, (b) CuVO_x_-2, and (c) CuVO_x_-4 electrodes after 2^nd^ cycle.

**Table S1.** Comparative electrochemical performance of the CuVO_x_-2 electrode with recently reported vanadium-based cathodes.

| **Cathode** | **Synthesis** | **Performance** | **Electrolyte** | **Ref.** |
| --- | --- | --- | --- | --- |
| FeVO | Hydrothermal | 70 mAh g^-1^ at 5 A g^-1^ | 1 M Zn(ClO_4_)_2_ | [1] |
| VOH | Hydrothermal | 225 mAh g^-1^ at 1 A g^-1^ | 2 M Zn(CF_3_SO_3_)_2_ | [2] |
| NaV_3_O_8_ | Coprecipitation | 221 mAh g^-1^ at 1 A g^-1^ | 1 M ZnSO_4_ | [3] |
| Ca_0.33_V_2_O_5_ | Hydrothermal | 354 mAh g^-1^ at 0.2 A g^-1^ | 3 M Zn(CF_3_SO_3_)_2_ | [4] |
| NH_4_V_4_O_10_ | Hydrothermal | 126 mAh g^-1^ at 0.2 A g^-1^ | 3 M Zn(CF_3_SO_3_)_2_ | [5] |
| Zn_0.25_V_2_O_5_ | Hydrothermal | 300 mAh g^-1^ at 0.05 A g^-1^ | 1 M ZnSO_4_ | [6] |
| Ba_1.2_V_6_O_16_ | Hydrothermal | 129 mAh g^-1^ at 5 A g^-1^ | 2 M ZnSO_4_ | [7] |
| A-V_2_O_5_-G | Solvothermal | 489 mAh g^-1^ at 0.1 A g^-1^ | 3 M ZnSO_4_ | [8] |
| CuVO_x_-2 | Coprecipitation | 519 mAh g^-1^ at 0.5 A g^-1^  220 mAh g^-1^ at 5 A g^-1^ | 3 M Zn(CF_3_SO_3_)_2_ | This work |

**References**

[1] Y. Luo, L. Wei, H. Geng, Y. Zhang, Y. Yang, C.C. Li. *ACS Appl. Mater. Interfaces* 2020, 12, 11753-11760.

[2] B. Ju, H.J. Song, H. Yoon, D.W. Kim. *Chem. Eng. J.* 420 (2021) 130528.

[3] F. Wan, L. Zhang, X. Dai, X. Wang, Z. Niu, J. Chen. *Nat. Commun.* 2018, 9, 1656.

[4] W. Zhou, M. Chen, A. Wang, A. Huang, J. Chen, X. Xu, C. Wong. *J. Energy Chem.* 2021, 52, 377-384.

[5] G. Yang, T. Wei, C. Wang. *ACS Appl. Mater. Interfaces* 2018, 10, 35079-35089.

[6] D. Kundu, B. D. Adams, V. Duffort, S. H. Vajargah, L. F. Nazar. *Nat. Energy* 2016, 1, 16119.

[7] X. Wang, B. Xi, X. Ma, Z. Feng, Y. Jia, J. Feng, Y. Qian, S. Xiong. *Nano Lett*. 2020, 20, 2899-2906.

[8] X. Wang, Y. Li, S. Wang, F. Zhou, P. Das, C. Sun, S. Zheng, Z. S. Wu. *Adv. Energy Mater.* 2020, 10, 2000081.
